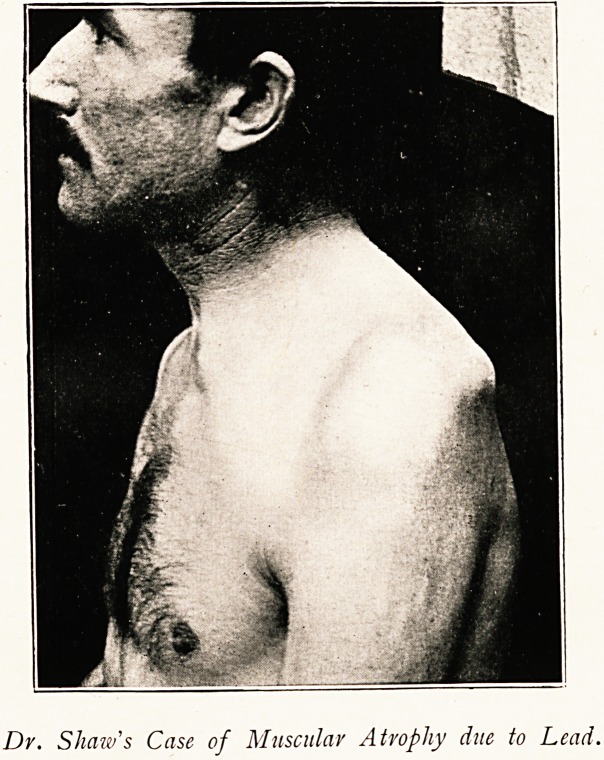# A Case of Muscular Atrophy Due to Lead

**Published:** 1901-06

**Authors:** J. E. Shaw

**Affiliations:** Physician to the Bristol Royal Infirmary; Professor of Medicine, University College, Bristol.


*Dr. Shaw's Case of Muscular Atrophy due to Lead.*

### A CASE OF MUSCULAR ATROPHY DUE TO LEAD.

BY

J. E. Shaw, M.B.,

*Physician to the Bristol Royal Infirmary; Professor of Medicine, University College, Bristol.*

J. A., æt. 42 years, farm labourer, was admitted to the Bristol Royal Infirmary on March 1st, 1901, complaining of weakness in his arms and legs. He stated that for about six weeks past he had noticed an increasing weakness in his hands and fore-arms, with pains about his shoulder-joints; these pains, he thought, were "rheumatic," and were the cause of his inability to move his shoulders. During this same time he had several attacks of colic, with constipation. He had been losing strength in his legs three or four weeks. He is accustomed to drink a good deal of cider.

*Condition on Admission.*—Looks in fair general health and nutrition. Arteries manifest decided sclerosis, with pulse of high tension; marked blue line on gums, tongue coated, bowels constipated, abdomen flat, liver not appreciably enlarged. Urine contains decided trace of albumen, but is not of abnormally low specific gravity.

*Nervous System: On Left Side.*—As will be seen in the plate, the deltoid, supra-spinatus, and infra-spinatus are almost completely atrophied. The portion of the trapezius attached to the clavicle (*ultimum moriens*) is in good development, the rest of the muscle having completely atrophied; the subscapularis is nearly all gone, and the latissimus dorsi weakened, though not completely paralysed. The clavicular fibres of the pectoralis major are quite wasted. The biceps and brachialis anticus are very much wasted, the residuum being toneless and flabby, so also is the supinator longus; the triceps, on the other hand, has quite escaped implication, and is of full size and possibly of full power. The muscles of the fore-arm present the condition usual in lead paralysis. The thenar and hypothenar muscles are much wasted, but not completely paralysed; the fingers cannot be flexed at the metacarpo-phalangeal joints by means of the interossei, which are wasted, and the lumbricales do not appear to have any power; the defective power of flexion and extension of the fingers, and perhaps also of abduction and adduction, may be increased by the state of the fore-arm muscles. Of the wasted muscles the mechanical irritability is much increased and "tendon reflexes" not obtainable. No response to faradic current can be obtained in the deltoid, biceps, supra- and infra-spinati, and supinator longus; the portion of the trapezius which remains responds readily, as does the triceps; the fore-arm extensors act very slightly, the flexors more readily. Some of the muscles, especially the infra-spinatus, are very tender on pressure; but no skin-anæsthesia or other sensory change is discoverable. Patient has no voluntary motor power over the shoulder-joint; possesses fair power of extension, but none of flexion, in the elbow-joint; none of extension, pronation, or supination at the wrist-joint; no power of extension of fingers, and marked loss of power in the proper muscles of the hand, as already noted.

*On Right Side* there is a general correspondence in condition, but to a lesser degree. The supra- and infra-spinati are rather wasted, the deltoid and clavicular fibres of the pectoralis major being of normal size apparently, though not of normal power; the biceps is of full volume, but decidedly flabby in tone, and the mechanical irritability much increased. The subscapularis is in better nutrition than upon the left side, and the latissimus dorsi apparently quite normal. The fore-arm muscles are much as upon the other side, but not quite so much affected. The thenar and hypothenar eminences are rather more affected than those of the left hand, the interossei apparently less so. To the faradic current the right supra- and infra-spinati are nearly as defective as those on the left side; but the deltoid, biceps and supinator longus give response to a strong current. Muscular hyperalgesia is most marked in the scapular muscles. There is apparently some general loss of electro-sensibility in affected areas, as the patient is not incommoded by a strong current applied to shoulders and upper arms. He is able to abduct slightly the arm from the side and raise the arm into the horizontal front position; he can also flex the elbow, but with less than normal power, though the biceps is not diminished in volume. The power of the fore-arm muscles is rather greater than upon the other side; in the hand he can abduct the fore-finger distinctly, and can oppose the tip of the thumb to the tip of the forefinger.

No affection of any muscle of face, eyes, lips, tongue, or palate. There is a general weakness of the legs, but no distinct paralysis or atrophy of any muscle in particular. No affection of the sphincters.

These cases of extensive muscular wasting and paralysis due to lead are well recognised, and are described in authoritative treatises, such as those by Oliver [1] and Gowers. [2] Dr. Shingleton Smith recorded a case resembling this one in some respects in *International Clinics* (6th series, vol. iii., p. 169); and quite recently Dr. D. J. McCarthy, in the *Philadelphia Medical Journal* (March 23rd, 1901, p. 574), describes a case due to lead and alcohol combined. Still, in this part of England we seldom see them.

It is clear that this clinical group of cases, though bearing a close external resemblance to one another in the affection of the shoulder group of muscles, are not all of the same fundamental pathology. In this particular case the rapid wasting, the marked loss of response to faradism, and the weakness of unatrophied muscles indicate a lesion in nerves rather than one located in the anterior cornua. Fibrillation also was non-existent; but no reliance must be placed upon the absence of that sign, significant as its presence often is.

[1] *Clifford Allbutt's System of Medicine*, vol. ii., 1897.

[2] *Diseases of the Nervous System*, vol. ii., 2nd ed., 1893, p. 950.

When last seen the patient had made considerable improvement in the power of the legs and fore-arms, and some improvement in the nutrition and power of the proper muscles of the hands; but many months, if not years, would be necessary, under the most favourable circumstances, for the restoration of the shoulder-girdle muscles.

---

### A CASE OF ARTIFICIAL ANUS FOLLOWING HERNIOTOMY.[1]

## Figures and Tables

**Figure f1:**